# Supplementary material for: Neuronal variability reflects probabilistic inference tuned to natural image statistics
Source: Nat Commun. 2021 Jun 15;12:3635. doi: 10.1038/s41467-021-23838-x (PMC8206154; doi:10.1038/s41467-021-23838-x)
Supplement: Supplementary file 3 — Reporting Summary [file 41467_2021_23838_MOESM3_ESM.pdf]

nature research

Corresponding author(s): Ruben Coen-Cagli

Last updated by author(s): Apr 9, 2021

Reporting Summary

Nature Research wishes to improve the reproducibility of the work that we publish. This form provides structure for consistency and transparency in reporting. For further information on Nature Research policies, see our Editorial Policies and the Editorial Policy Checklist.

Statistics

For all statistical analyses, confirm that the following items are present in the figure legend, table legend, main text, or Methods section.

n/a	Confirmed	
☐	☒	The exact sample size (*n*) for each experimental group/condition, given as a discrete number and unit of measurement
☐	☒	A statement on whether measurements were taken from distinct samples or whether the same sample was measured repeatedly
☐	☒	The statistical test(s) used AND whether they are one- or two-sided<br>*Only common tests should be described solely by name; describe more complex techniques in the Methods section.*
☐	☒	A description of all covariates tested
☒	☐	A description of any assumptions or corrections, such as tests of normality and adjustment for multiple comparisons
☐	☒	A full description of the statistical parameters including central tendency (e.g. means) or other basic estimates (e.g. regression coefficient) AND variation (e.g. standard deviation) or associated estimates of uncertainty (e.g. confidence intervals)
☐	☒	For null hypothesis testing, the test statistic (e.g. *F*, *t*, *r*) with confidence intervals, effect sizes, degrees of freedom and *P* value noted<br>*Give P values as exact values whenever suitable.*
☒	☐	For Bayesian analysis, information on the choice of priors and Markov chain Monte Carlo settings
☒	☐	For hierarchical and complex designs, identification of the appropriate level for tests and full reporting of outcomes
☐	☒	Estimates of effect sizes (e.g. Cohen's *d*, Pearson's *r*), indicating how they were calculated

Our web collection on statistics for biologists contains articles on many of the points above.

Software and code

Policy information about availability of computer code

Data collection	Blackrock Microsystem and RippleNeuron were used for collecting spike data. EyeLink 1000 eye tracker for awake animals. OpenGL Expo (V1.5; https://sites.google.com/a/nyu.edu/expo/) was used to display visual stimuli and pair stimulus timings with physiological data.
Data analysis	We sorted waveforms manually using Plexon Offline Sorter (V3). Spike times and stimuli parameters were then extracted using a Matlab script (V 2016a). Data was analyzed using custom code written in Julia 1.5 (https://julialang.org)<br>Model simulations were performed with custom code written in Julia (V1.5) and Stan (https://mc-stan.com, CmdStan V2.20)<br>Code for model simulations and data analysis is available without restrictions on GitHub (https://github.com/rubencoencagli/festa-et-al-2020 , DOI: 10.5281/zenodo.4710150).

For manuscripts utilizing custom algorithms or software that are central to the research but not yet described in published literature, software must be made available to editors and reviewers. We strongly encourage code deposition in a community repository (e.g. GitHub). See the Nature Research guidelines for submitting code & software for further information.

Data

Policy information about availability of data

All manuscripts must include a data availability statement. This statement should provide the following information, where applicable:

- Accession codes, unique identifiers, or web links for publicly available datasets
- A list of figures that have associated raw data
- A description of any restrictions on data availability

Source data are provided with this paper. Data for Figure 2c and Supplementary Figure S6B are publicly available on the CRCNS data sharing site crcns.org. Data for the other figures can be found at https://doi.org/10.5281/zenodo.4710066 . Natural images used to train the GSM models are publicly available in the BSDS500 database (https://github.com/BIDS/BSDS500).

Field-specific reporting

Please select the one below that is the best fit for your research. If you are not sure, read the appropriate sections before making your selection.

☒ Life sciences ☐ Behavioural & social sciences ☐ Ecological, evolutionary & environmental sciences

For a reference copy of the document with all sections, see nature.com/documents/nr-reporting-summary-flat.pdf

Life sciences study design

All studies must disclose on these points even when the disclosure is negative.

Sample size	The effects reported are based on the following sample sizes (number of independent neurons), which are well in line with the standards of the field when studying single-neuron response properties (e.g. Henaff et al 2020 Nature Communications https://doi.org/10.1038/s41467-020-15533-0): Fig. 2C, n=261, from 3 anesthetized macaques; Fig 2F and Table 1, n=105, from one awake animal and n=229, from 3 anesthetized macaques; Fig. 3, n=71, from 2 awake macaques.
Data exclusions	The exclusion criteria are discussed in detail in the Methods, Section "Characterization of neuronal responses and inclusion criteria". The criteria were defined primarily to ensure that neurons were visually driven and there was measurable response to at least some of the stimuli presented, and that the neurons' RFs were well centered on the stimuli.
Replication	Reproducibility of measured single-neuron quantities was verified across recording sessions, animals and stimulus classes (Figures 2,3; Table 1). All attempts were successful.
Randomization	Presentation of different stimulus conditions was randomized.
Blinding	N/A this study does not report a comparison between different populations.

Reporting for specific materials, systems and methods

We require information from authors about some types of materials, experimental systems and methods used in many studies. Here, indicate whether each material, system or method listed is relevant to your study. If you are not sure if a list item applies to your research, read the appropriate section before selecting a response.

Materials & experimental systems

n/a	Involved in the study	
☒	☐	Antibodies
☒	☐	Eukaryotic cell lines
☒	☐	Palaeontology and archaeology
☐	☒	Animals and other organisms
☒	☐	Human research participants
☒	☐	Clinical data
☒	☐	Dual use research of concern

Methods

n/a	Involved in the study	
☒	☐	ChIP-seq
☒	☐	Flow cytometry
☒	☐	MRI-based neuroimaging

Animals and other organisms

Policy information about studies involving animals; ARRIVE guidelines recommended for reporting animal research

Laboratory animals	Data recorded from 5 male adult macaque monkeys (Macaca fascicularis) age 3-8 years.
Wild animals	The study did not involve wild animals.
Field-collected samples	The study did not involve field-collected samples.
Ethics oversight	All procedures were approved by the Albert Einstein College of Medicine and followed the guidelines in the United States Public Health Service Guide for the Care and Use of Laboratory Animals.

Note that full information on the approval of the study protocol must also be provided in the manuscript.
